# CNAReporter: a GenePattern pipeline for the generation of clinical reports of genomic alterations

**DOI:** 10.1186/1755-8794-3-11

**Published:** 2010-04-09

**Authors:** Yuri Kotliarov, Serdar Bozdag, Hangjiong Cheng, Stefan Wuchty, Jean-Claude Zenklusen, Howard A Fine

**Affiliations:** 1 Neuro-Oncology Branch, National Cancer Institute, National Institutes of Neurological Disorder and Stroke, National Institutes of Health, 37 Convent Drive, Bethesda, MD 20892, USA; 2 NCI Center for Biomedical Informatics and Information Technology, National Cancer Institute, National Institutes of Health, 2115 E Jefferson St., Rockville, MD 20852, USA

## Abstract

**Background:**

Genomic copy number alterations are widely associated with a broad range of human tumors and offer the potential to be used as a diagnostic tool. Especially in the emerging era of personalized medicine medical informatics tools that allow the fast visualization and analysis of genomic alterations of a patient's genomic profile for diagnostic and potential treatment purposes increasingly gain importance.

**Results:**

We developed CNAReporter, a software tool that allows users to visualize SNP-specific data obtained from Affymetrix arrays and generate PDF-reports as output. We combined standard algorithms for the analysis of chromosomal alterations, utilizing the widely applied GenePattern framework. As an example, we show genome analyses of two patients with distinctly different CNA profiles using the tool.

**Conclusions:**

Glioma subtypes, characterized by different genomic alterations, are often treated differently but can be difficult to differentiate pathologically. CNAReporter offers a user-friendly way to visualize and analyse genomic changes of any given tumor genomic profile, thereby leading to an accurate diagnosis and patient-specific treatment.

## Background

Genomic copy number alterations are widely associated with a broad range of human diseases [1]. In general, tumors [2] have genomic abnormalities that are largely characterized by copy number alterations. Specifically, amplifications, deletions and allelic imbalances are hallmarks of human gliomas [3,4]. Such genomic data offer important biological insights into the pathogenesis of the disease and might serve as valuable clinical and diagnostic tool. By classifying patients into more homogeneous tumor groups, genomic alteration data also might allow the enrichment of patient subpopulations with genetic targets that are more likely to respond to specific molecularly targeted therapy.

Copy number analysis for a single patient usually include several major steps, such as (1) raw data processing with normalization and calculation of log_2 _ratios of probe intensities in tumor compared to either a reference sample or reference set - the values representing copy numbers; (2) smoothing and segmentation of copy numbers followed by selection of areas of copy number alterations (CNA); (3) if available, analysis of loss-of-heterozygosity (LOH) and (4) visualization of the CNA/LOH profile.

Many tools for the analysis of copy number profiles have been developed by the scientific community and are often freely available and cover all steps of analyses such as Affymetrix CNAT [5], CNAG [6], dChipSNP [7], ArrayFusion [8], perl-based PennCNV [9], as well as several R/Bioconductor packages like aroma.affymetrix [10] and SNPchip [11]. Most of these software packages, however, require that the user has substantial bioinformatics knowledge and computer/programming skills. The output is generally an interactive browser of genomic profiles and/or exported figures/text files. With an ever-increasing demand for patient-specific genomic data by clinical researchers and clinicians, however, there is a great need for analyses tools and output formats that individuals without computational expertise can utilize to generate such information.

Our goal, therefore, was to create an easy to use tool for non-sophisticated users who would want a "snapshot" of the genomic profile for a particular tumor/tissue sample from the raw microarray data and to obtain that data in an easy to understand printable format suitable for clinical trial study charts or medical records. Based on the widely used GenePattern framework [12], we developed CNAReporter, a reporting tool that interprets experimental measurements from high resolution Human Mapping GeneChip arrays (Affymetrix Inc., Santa Clara, CA) [13]. Providing statistical treatment of such data, CNAReporter determines and annotates regions of genomic alterations in a sample and summarizes results in a printable PDF-file. Specifically, we show the usefulness of CNAReporter as a clinical tool that supports the accurate diagnosis and treatment of patients with primary brain tumors.

## Implementation

### Data, Workflow and User Interface

**Figure 1:**
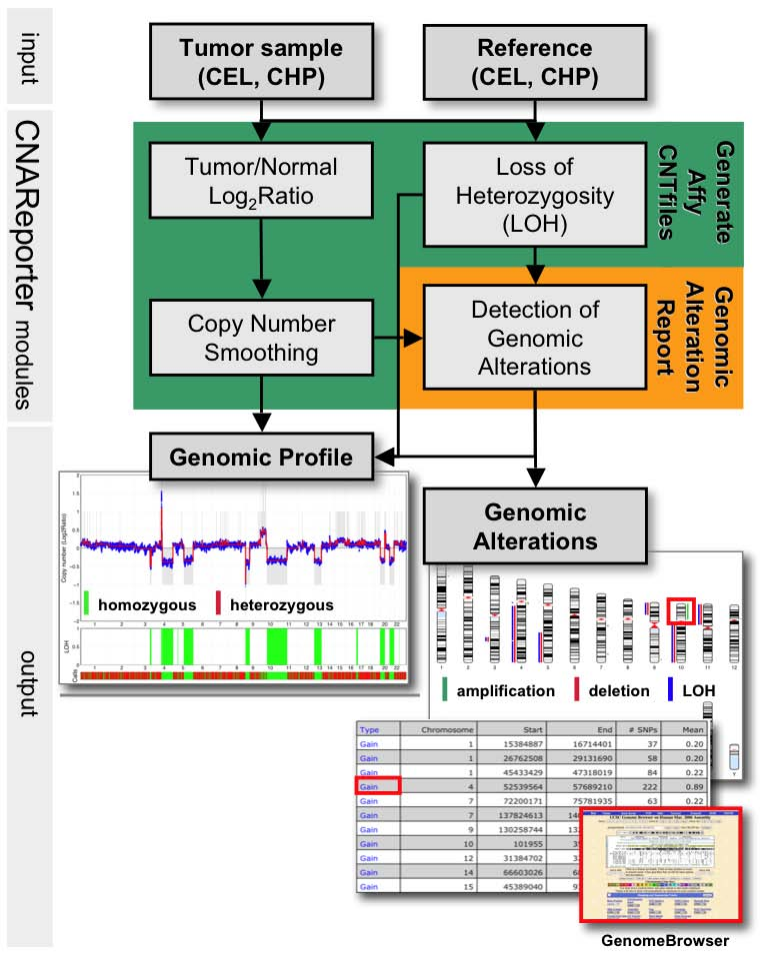
**Workflow of CNAReporter, describing sample input files, functions of CNAReporter modules and output examples**. Specifically, areas of significant genomic change are tabulated in a PDF-file, characterized by its corresponding type, such as chromosomal gain, loss and allelic imbalances and linked to UCSC Genome Browser [20]. Furthermore, the patient specific report includes a genomic profile that provides raw and smoothed profiles of log2 ratios of sample to reference intensities representing copy numbers, areas of loss-of-heterozygosity (LOH) and genomic calls. These areas are finally labelled in a plot of chromosomal changes that are optionally available for each chromosome separately.

CNAReporter calls for a matched pair of patient-specific tumor and germline reference CEL and CHP files (Figure 1), which are usually generated by Affymetrix GTYPE or Genotyping Console software [14,15].

We designed CNAReporter as a GenePattern 3.0 pipeline [12], consisting of two modules (Figure 1). From paired input data, the module *GenerateAffyCNTfiles *calculates copy numbers (CN), provides smoothed CN-profiles and calculates LOH status of genomic locations. All results are stored in standard Affymetrix CNT files. The module was written in Perl as a wrapper for platform corresponding binary executables of the Affymetrix DevNet Tools copy-number pipeline [16], allowing the on-the-fly generation of all intermediate files. Details about the corresponding algorithms can be found in [5].

Reading generated CNT-files, the module *GenerateAlterationReport *determines CNA and LOH areas. The final output is a printable PDF-file that provides a table of altered genomic areas and graphic visualizations as a genomic profile and chromosome plot (Figure 1). The module was implemented in MATLAB (The Mathworks, Inc., Natick, MA) requiring the Bioinformatics Toolbox to create chromosome plots and using a Perl script to generate the final PDF report.

CNAReporter provides a user interface to a standard GenePattern pipeline, allowing the input of the aforementioned Affymetrix-specific files. Advanced options include the selection of thresholds for the detection of CNA and LOH as well as the ability to plot genomic profiles for individual chromosomes.

CNAReporter runs on all platforms that GenePattern, MATLAB and Affymetrix tools support, including Windows, Mac, Linux and Sun Solaris. Currently supported arrays include Affymetrix 500K, 100K and 10K human mapping arrays. Since the latest SNP5 and SNP6 arrays require different algorithms to estimate copy numbers, their support will be added in the future.

### Determination of copy numbers

Copy numbers are calculated using Affymetrix Copy Number Analysis Tool (CNAT 4), a set of command line programs. We allow filtering out SNPs with large PCR fragment length (MaxFragSize parameter, 600 bp by default) to support samples with partially degraded DNA [17]. For standard fresh-frozen samples this parameter can be set to 0 to include all SNPs. After probe-level normalization and summarization, calculated log_2_-tranformed ratios are used to estimate raw copy numbers (CN). Using a Gaussian approach, raw SNP profiles are smoothed (>500 kb window by default) and segmented by a Hidden Markov Model approach [18,19]. Raw and smoothed copy numbers are saved in an Affymetrix-based CNT file.

### Determination of loss of heterozygosity (LOH)

LOH calls for each SNP are determined by comparing corresponding genomic calls in the tumor and the germline sample, provided that SNPs are heterozygous in the reference sample. Specifically, we use the LOH algorithm as implemented in CNAT 4 [5]. LOH values are also segmented utilizing a Hidden Markov Model and saved in an Affymetrix-based CNT file.

### CNA areas

Utilizing copy numbers and LOHs that characterize the underlying tumor sample by individual SNPs, CNAReporter applies three threshold parameters to define areas of CNAs. Increasing the absolute values of those parameters makes detection more conservative, thereby, decreasing the number of false positive areas but increasing the possibility of missing real changes. In addition, neighboring LOH areas are combined if they are within a certain threshold distance from each other (set by LOHMergeThreshold parameter, 2 Mbp by default). Lowering this parameter may cause splitting of LOH areas due to errors in genotyping calls. Default thresholds are determined empirically from the analysis of other brain tumor samples.

## Results

### Patient examples

Tissue from fresh frozen tumor specimens and resultant data were collected under an NCI-IRB (FWA # 00005897/IRB# 00000001)-approved protocol (NCI#:02C0140). Informed consent was obtained from each patient and documented in the medical records. Specimens and data were de-identified to comply with patients' privacy rules.

**Figure 2:**
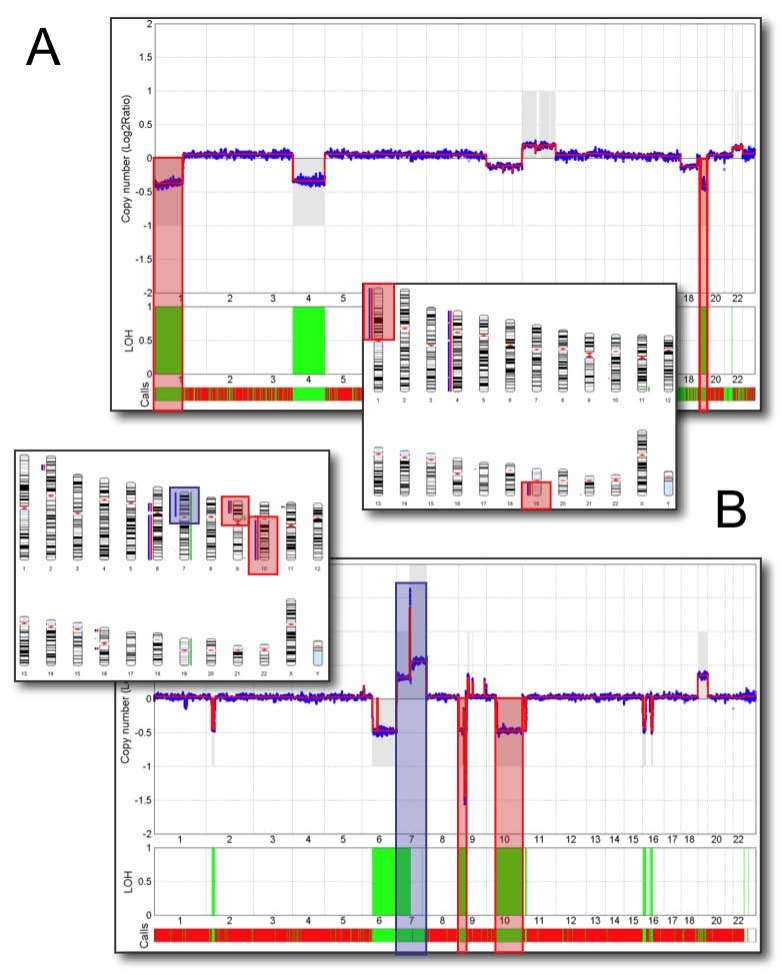
**Diagnostic examples**. In **(A) **we show a patient sample predominantly with strong deletions on chromosome 1, 4 and 19. The prevalence of a deletion and loss-of-heterozygosity (LOH) of 1p and 19q areas indicates the presence of an oligodendroglioma (red shaded areas in the genomic profile and chromosome plot). In **(B)**, a patient sample shows a variety of copy number alterations. While the 1p/19q deletions are missing, we find a large deletion with LOH on chromosome 10, homozygous deletion on chromosome 9 (red shaded areas) and amplification of chromosome 7 (blue shaded areas), changes that are prototypic for a glioblastoma.

Routinely, we analyse genomic profiles of glioma patients with CNAReporter. Specifically, we extract and hybridize DNA from patient tumor samples to the Affymetrix 500K SNP chips [13] using methods previously reported [17]. As representative examples, that demonstrate the usefulness of CNAReporter for diagnostic and treatment purposes, we show genome analyses of two patients with distinctly different CNA profiles. Given their general resistance to conventional therapy glioblastomas are the most common and malignant type of gliomas and are treated aggressively with a potentially toxic combination of high-dose cranial radiotherapy and chemotherapy. A much less common type of malignant glioma, called an anaplastic/malignant oligodendroglioma, can occasionally be very difficult to distinguish from a glioblastoma by standard pathological criteria. Clinically the distinction between these two types of gliomas, however, is utterly important since oligodendrogliomas, harbouring the characteristic chromosome 1p and 19q deletion, are often significantly more sensitive to treatment than glioblastomas. Thus, patients with oligodendrogliomas can often be treated with drug therapy alone thereby sparing them the potential long-term neuro-cognitive toxicity from brain radiation. In Figure 2A, we observe clear deletions of chromosomes 1p and 19q, an area that is typical for patients with an oligodendroglioma. This is in clear contrast to the genomic profile of the tumor seen in Figure 2B where the 1p/19q deletion is absent whereas a strong amplification of chromosome 7 (with a high peak corresponding to the epidermal growth factor receptor), deletion of chromosome 10 and homozygous deletion on chromosome 9 are seen; all highly characteristic of a glioblastoma. Thus, through ready access to CNAReporter, a clinician would be able to offer the appropriate treatment to both patients, something that may not have occurred if treatment were based purely on standard histopathological criteria, as is currently the standard of care.

## Conclusions

We introduced CNAReporter, a user-friendly, integrated tool that allows the quick analysis and visualization of chromosomal alterations. In particular, CNAReporter provides detailed high-quality reports of genomic alterations in a printable format, allowing our application to be used as a standard tool for clinical diagnostics and decision-making. Specifically, we use CNAReporter routinely for the analysis of genomic alterations of brain tumor tissues, potentially allowing us to make objective tumor subtype diagnoses, stratify patients into biologically more homogeneous tumor subgroups for clinical trials and select patient-specific treatments based on objective genomic data. We designed CNAReporter as a pipeline in GenePattern environment, a well-known open-source web-based framework that supports multiple platforms. Once properly installed on a server, GenePattern does not require additional software to be installed on the user's computer and can be accessed from any site with only a web browser. The GenePattern framework provides security, job management, uniform interface, relative ease of customization and integration with other developers' tools. Currently, GenePattern already has several modules for SNP analysis in its repository, such as the preprocessing SNPFileCreator module (which does not implement paired analysis) and GISTIC for chromosomal aberrations discovery in multi-sample datasets [4]. We believe our tool would be a significant addition to this suite. Due to its open architecture, CNAReporter can easily be further developed in an open-source sense and integrated into other systems for genomic analysis.

## Availability and Requirements

CNAReporter with installation and usage instructions as well as all required files can be downloaded from http://gforge.nci.nih.gov/projects/cnareport.

The program is available for Linux/Unix, Mac and Windows operating systems, and requires MATLAB 2007b (or later) with Bioinformatics Toolbox, Perl 5 (including CRAN libraries) and GenePattern 3.0 (or later).

## Abbreviations

CN: (DNA) copy number; CNA: copy number alteration; LOH: loss of heterozygosity; SNP: single nucleotide polymorphism; (M)bp: (Mega) base pairs.

## Authors' contributions

YK, JCZ, SW and HF formulated the requirements of the tool. YK designed the pipeline. YK, SB and HC implemented the tool. HF coordinated the study. All authors read and approved the final manuscript.

## Pre-publication history

The pre-publication history for this paper can be accessed here:

http://www.biomedcentral.com/1755-8794/3/11/prepub
